# Post-reproductive parthenogenetic pea aphids (*Acyrthosiphon pisum*) are visually identifiable and disproportionately positioned distally to clonal colonies

**DOI:** 10.7717/peerj.2631

**Published:** 2016-10-26

**Authors:** Erik T. Saberski, Julia Daisy Diamond, Nathaniel Fath Henneman, Daniel A. Levitis

**Affiliations:** 1 Department of Biology, Bates College, Lewiston, Maine, United States; 2 Department of Botany, University of Wisconsin-Madison, Madison, Wisconsin, United States

**Keywords:** Aphid development, Menopause, Reproductive aging, Insect demography, Evolutionary demography, Post-reproductive lifespan

## Abstract

The role of kin-selection in the evolution of post-reproductive life is controversial. While anthropological and demographic studies strongly suggest that humans and a few other species experience kin selection for significant post-reproductive survival, these results are necessarily correlational. Understanding could therefore be advanced by the development of a globally available, field and laboratory tractable experimental model of kin-selected post-reproductive survival. In only one invertebrate (*Quadrartus yoshinomiyai*, a gall-forming aphid endemic to Japan) have individuals too old to reproduce been shown to be both numerous in natural habitats and able to help close relatives survive or reproduce. Pea aphids, (*Acyrthosiphon pisum*), common, tractable organisms, frequently outlive their reproductive ages in laboratories, live in tight interacting groups that are often clonal, and therefore should be evaluated as potential model organisms for the study of adaptive post-reproductive life. The first major step in this process is to identify an optimal method for assessing if a parthenogenetic adult is post-reproductive. We evaluated three methods, relying respectively on isolation in clip cages, visual examination for embryonic eyespots, and dissection. In every case each method identified the same individuals as reproductive versus post-reproductive. While the clip-cage method requires a multi-day wait to produce data, and dissection is inevitably fatal, the eyespot method is quick (under one minute per individual) easy, and non-invasive. This method makes it possible to accurately assess the post-reproductive status of a large number of parthenogenetic pea aphids. We demonstrate the usefulness of the eyespot method in showing that while reproductively valuable adults tend to place themselves near the centers of clonal colonies, less valuable post-reproductive adults are more often at or beyond the edges of colonies. These encouraging early results provide both impetuous and aid for further investigations into the post-reproductive lives of pea aphids.

## Introduction

Humans were long considered to be the only species in which many individuals lived well past the age at which they stopped reproducing (Hawkes et al., 1998; Peccei, 1995). Evolutionary explanations for human post-reproductive demography (Hawkes & Coxworth, 2013; Hill & Hurtado, 1991; Pavard, Metcalf & Heyer, 2008) often rely on kin-selection arguments: alleles that favor survival past the age of reproductive cessation can be selectively advantageous if they allow older individuals to continue helping younger kin (who carry the same alleles) to survive and reproduce. Living human grandmothers can enhance fitness outcomes of their kin (Lahdenperä et al., 2004), and humans are the only primates known to experience significant post-reproductive survival outside of captivity (Alberts et al., 2013; Levitis, Burger & Lackey, 2013; Levitis & Lackey, 2011).

A great taxonomic diversity of organisms shown have the capacity for post-reproductive life (Cohen, 2004; Levitis, Burger & Lackey, 2013), but for surprisingly few of these has any selective benefit to outliving one’s own fertility been demonstrated. A wide variety of evolutionary explanations, only some of which are selective, and many of which have little to do with humans, have therefore been proposed (recently reviewed by Croft et al. (2015)). The evolutionary study of post-reproductive life has therefore expanded from a fairly strict focus on human socioecology (Hawkes, O’Connell & Blurton Jones, 1989), to include comparisons of humans to other primates (Hawkes & Coxworth, 2013; Judge & Carey, 2000), comparisons across mammals (Cohen, 2004), studies focusing on numerous other species (vertebrates and invertebrates), and comparative studies that consider the full variety of species for which data are available (Croft et al., 2015; Levitis, Burger & Lackey, 2013). Such comparative thinking gives us a framework in which to understand human post-reproductive life, but also it allows us to make post-reproductive survival a topic for evolutionary inquiry that need not be centered on humans. The central question in this context is why evolution should lead to life-histories that include survival by post-reproductive individuals. Women are the most thoroughly examined example of the phenomenon to be explained, but not necessarily central to the question.

Even the study of specifically kin-selected post-reproductive life has expanded well beyond female primates. In the last decade, strong arguments for adaptive post-reproductive life-stages has emerged in men (Vinicius, Mace & Migliano, 2014; Vinicius & Migliano, 2016) and resident killer whales (Brent et al., 2015; Foster et al., 2012). Less definitively, two other species of toothed whales (false killer whales (Photopoulou et al., 2016) and short-finned pilot whales (Kasuya & Marsh, 1984; Marsh & Kasuya, 1984)) as well as both extant species of elephants (Lahdenperä, Mar & Lummaa, 2014; Lee et al., 2016) have been proposed as having significant post-reproductive survival, and socioecological system that could select for the survival of post-reproductive females.

Moving beyond vertebrates, *Quadrartus yoshinomiyai,* a Japanese gall-forming aphid (Uematsu et al., 2010; Uematsu, Shimada & Shibao, 2013) has a high representation of post-reproductive females with an important role in the success of their kin group. In *Q. yoshinomiyai*, a clonal colony grows inside a sealed gall. When the gall is opened to allow dispersal, the risk that a predator will enter the gall increases. The colony defends itself against this by having a significant proportion of its adult females break down their reproductive organs and instead fill their abdominal cavities with enlarged wax glands. These post-reproductive females position themselves near the gall entrance (Uematsu, Shimada & Shibao, 2013) with their abdomens filled with a sticky wax. When a predator attempts to enter the gall, post-reproductive aphids attack to cover the predator with wax, often killing themselves in the process. Experimental removal of these defenders leads to predators more successfully entering and hunting inside (Foster, 2010; Uematsu et al., 2010). Gall defense (most often by pre-reproductive nymphs or non-reproductive soldiers) has been shown to improve selective outcomes for the clonal group in several aphid species (Hattori, Kishida & Itino, 2013; Itô, 1989).

One key tool still largely lacking from the comparative study of adaptive post-reproductive life is a widely available experimental system. Since the ecology and evolution of humans, whales and elephants populations cannot feasibly or ethically be studied in the laboratory, various controversies persist about the importance of selection in their post-reproductive lives, (e.g., Robeck et al., 2016) and likely will do so indefinitely. Aphids could provide a valuable experimental system for understanding the adaptive value of post-reproductive life. *Q. yoshinomiyai* is, in several respects a major improvement over large mammals, and promises to greatly advance our understanding of adaptive post-reproductive life. However, other aphids are even more experimentally tractable and available globally. As *Q. yoshinomiyai* is the only invertebrate in which adaptive post-reproductive life has been documented, we propose that other aphid species, particularly those with mutually beneficial clonal aggregations, should be examined for adaptive post-reproductive life stages.

A likely species to examine in this respect is the pea aphid (*Acyrthosiphon pisum* Harris). Pea aphids are widely available, the subject of a large and diverse body of previous and ongoing research (Brisson & Stern, 2006) and simple to raise and care for. A review of the literature reveals that authors have often noticed (Davis, 1915; Frazer, 1972; Mondor & Roitberg, 2003) and occasionally speculated about the function (Kidd & Tozer, 1985; Laughton, Fan & Gerardo, 2014) of the length of post-reproductive life in the pea aphid (*Acyrthosiphon pisum* Harris). Pea aphids are facultative parthenogens with complex life cycles, but clonal colonies are dominated and perpetuated by individuals that reproduce only asexually. These parthenogenetic pea aphids are born with a complete stock of embryos; when it is depleted, they are definitively post-reproductive. For a recent review on the life stages and development of pea aphids, see Schmidtberg & Vilcinskas (2016).

A key consideration in our search for a model species for adaptive post-reproductive survival is that it lives in aggregations of closely related individuals where inclusive fitness strongly influences behavior. Pea aphids live in colonies (i.e., aggregations on part of a single plant) that grow through parthenogenetic reproduction. Although in some cases these aggregations may contain more than one clonal lineage, relatedness within pea aphid colonies is generally high (Mondor & Messing, 2007). While aggregation can have benefits even in the absence of kin (Hamilton, 1971), a wide variety of behaviors benefiting clone mates in aggregations of pea aphids have been documented, including adaptive suicide (McAllister, Roitberg & Weldon, 1990), increased tolerance for cannibalistic feeding (Cooper, Desjonqueres & Leather, 2014), and scent-marking predators (Mondor & Roitberg, 2004). Aphids at the edges of colonies are at the highest risk of attack (Duff & Mondor, 2012; Obata, 1986), and when attacked will mark the attacker with alarm pheromone, effectively decreasing the risk for clone mates (Mondor & Roitberg, 2004).

Although parthenogenetic pea aphids have all of these advantages, it is not yet proven that they have an evolved post-reproductive life stage. Indeed, the behavior and field ecology of post-reproductive pea aphids remain largely unstudied. If post-reproductive individuals make up a significant proportion of natural pea aphid populations and have a meaningful positive net effect on their relatives’ fitness, then they will be an excellent experimental model for studying kin-selected post-reproductive life. The present study aims to lay the groundwork for testing these key points. Our specific goals are twofold. First, we aim to perfect a method for distinguishing post-reproductive parthenogentic pea aphids from younger adults. We test three methods of determining post-reproductive status against each other to determine their accuracy and logistical desirability. We demonstrate a method that is reliable, easy, fast and non-invasive.

We then employ that method to achieve our second goal: testing the prediction that post-reproductive individuals, being of low reproductive value to the group, tend to occupy the edges of colonies, where predation risk is predicted to be higher, and where defensive activities would most likely be needed. This prediction arises from two sources. Duff & Mondor (2012) find that reproducing pea aphid adults, being of high reproductive value, move to the center of the colony, while their nymphs, being of lower reproductive value (because they are more likely to die before having the opportunity to reproduce) are at the exposed edges of the colony. Following Duff and Mondor’s logic, post-reproductive individuals are of the lowest reproductive value (zero), and should take the most exposed posts. Uematsu, Shimada & Shibao (2013) find that post-reproductive *Q. yoshinomiyai* move toward the gall openings, where defense from predators is most often needed, while reproductive members of the same clones move away from the opening into the safety of the gall. Post-reproductive pea aphids could best serve some defense function if they move to the edges of the colony. While evidence for post-reproductive individuals being concentrated away from the center of colony would not prove that they are protecting the colony, knowing that they do so would be a useful early step in exploring possible adaptive roles for post-reproductive pea aphids.

## Materials and Methods

### Study population

A single wild *Acyrthosiphon pisum* was obtained from a bean plant at Fresh Start Farms, an organic farm in Lisbon, Maine and allowed to reproduce parthenogenetically in the laboratory on *Vicia faba* (fava bean plants) grown from seed (Jung Seed Company, Randolph, WI, USA). The population was founded from one individual reproducing clonally. While pea aphids come in both green and red morphs, this population was entirely green. We kept the population of aphids at 22 ± 3 °C. Temperatures inside clip cages were within 0.4 °C of room temperatures. We placed the plants 20 cm below fluorescent greenhouse lights with a 14/10 light/dark cycle.

### Methods testing for identifying post-reproductive individuals

**Figure 1:**
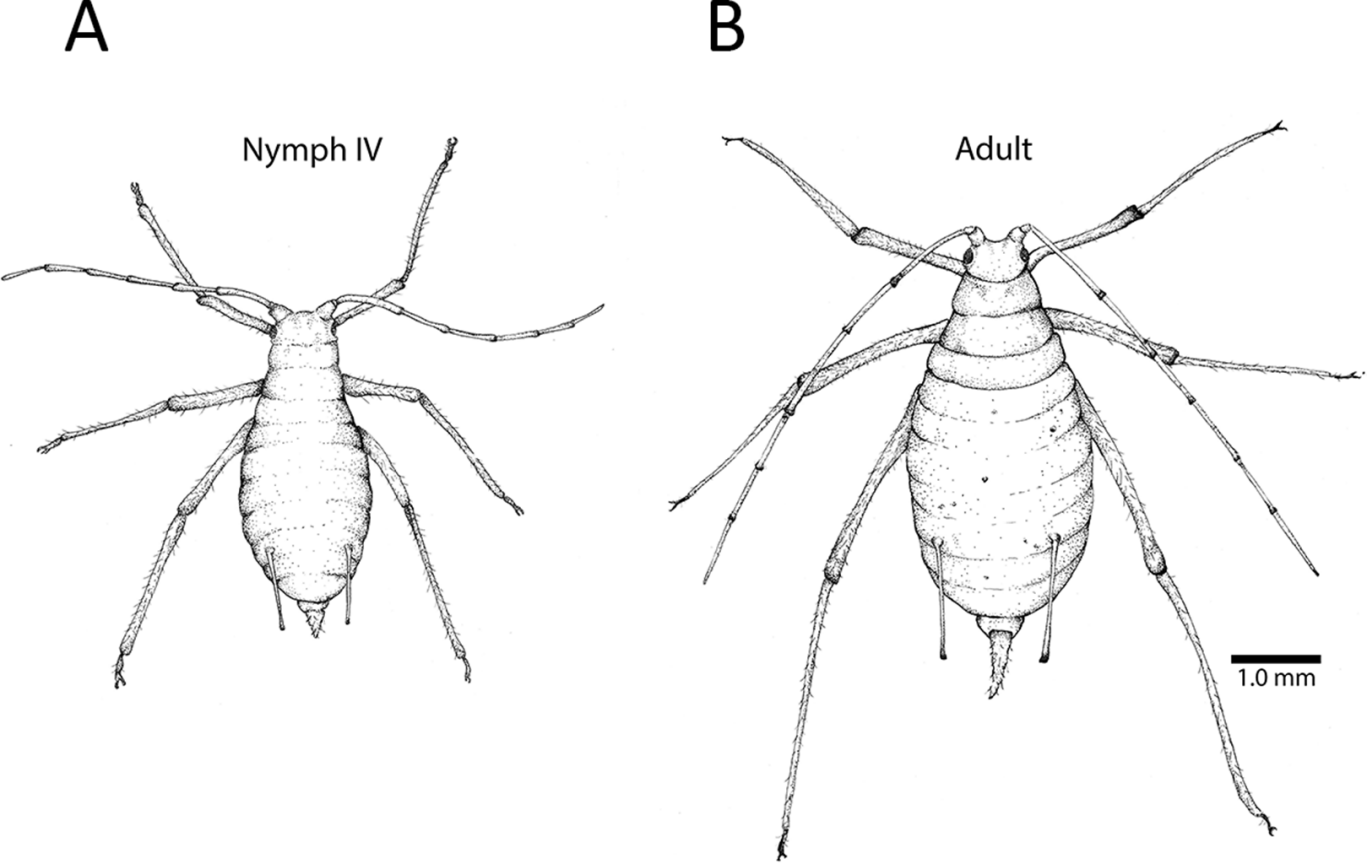
Fourth instar nymph and apterous adult. (A) The fourth instar nymph is distinguishable from the (B) wingless adult based on several morphological features, including the adult’s elongated cauda (the tail-like extension at the end of the abdomen). This adult has embryos developing in her ovaries, and their pigmented eyes are visible as spots in her abdomen. Depending on lighting, and the size and position of an embryo, one or both eyes may be visible. Illustration by Julia Diamond.

All focal individuals in our methods-development experiments were apterous (wingless) parthenogenetic adults. They were gathered from our stock population by gently shaking infested leaves over a petri dish. Care was taken to gather adults from multiple plants. Individual adults were then carefully picked from the dish using a fine paintbrush. Adults were easily distinguished from late nymphs (Instar IV) based on morphology (Fig. 1). We maintained up to 20 individuals in clip cages at a time, repeating the experiment five times for a total of 57 focal individuals, excluding six that escaped or died prior to data collection.

**Figure 2:**
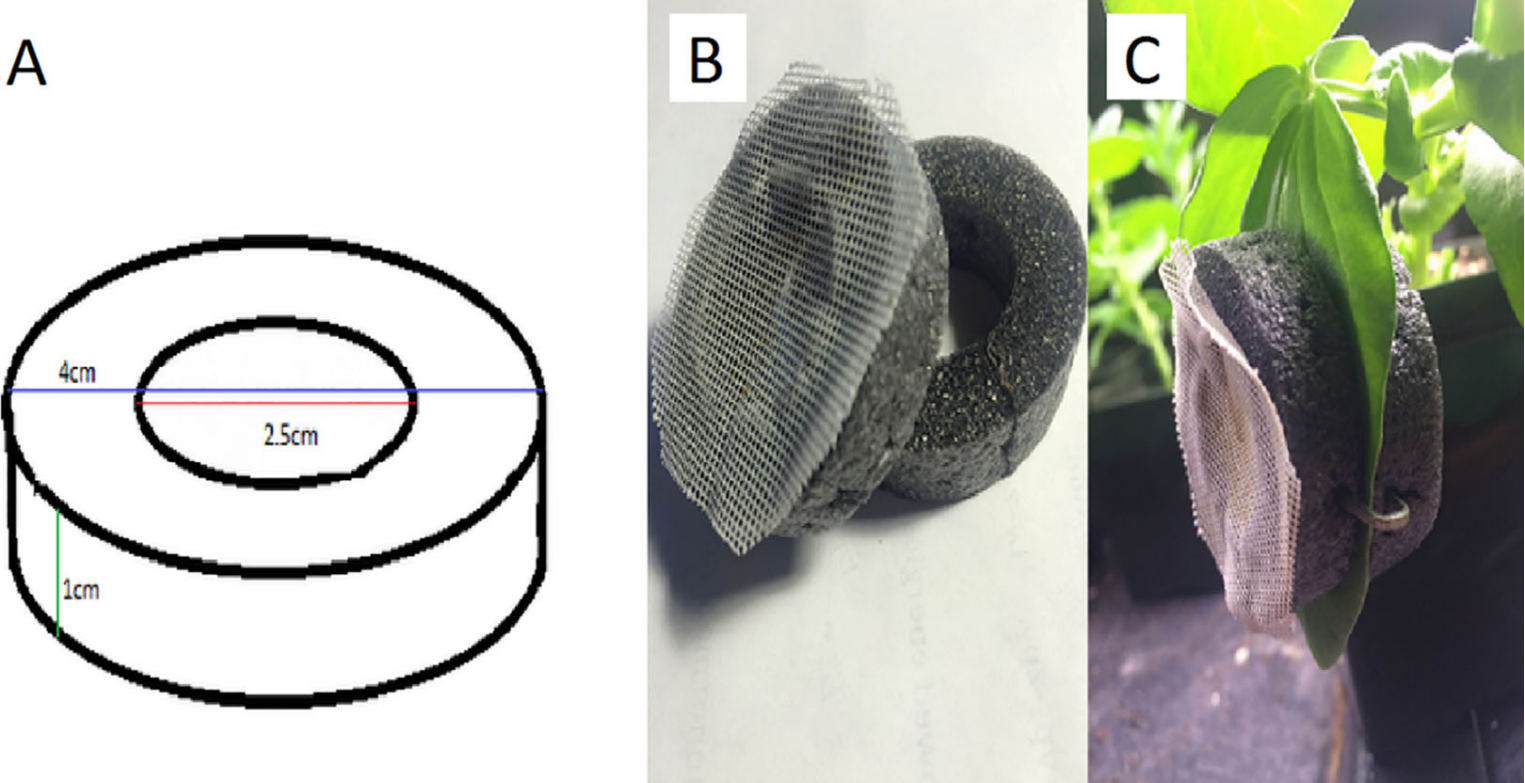
The design and use of a clip-cage. (A) We used rings of foam pipe insulation with the indicated dimensions, (B) glued mesh to half the rings and (C) used three staples equally spaced around the rings to attach the two parts of the clip cage to the leaf. Mesh occasionally begins to peel and needs to be re-glued. Photograph by Erik Saberski.

We evaluated three separate methods for assessing reproductive status of wingless parthenogentic adult pea aphids, beginning with two pre-existing methods available for determining whether a pea aphid is post-reproductive. The first was to isolate an individual and regularly remove her offspring (Gange, Bower & Brown, 1999). It is known that aphids generally reproduce every day when they are reproductive, with occasional gaps, particularly near the end of reproductive life. They produce an average of 72 offspring over two to three weeks (Trionnaire et al., 2008). Thus, once no offspring appear for four days in a row, one can conclude that the aphid is post-reproductive. We isolated pea aphids by placing them inside clip-cages (Fig. 2) and attaching them to the leaves of bean plants.

The second method of determining whether an aphid is post-reproductive requires no waiting, but is fatal: dissecting the aphid to observe if she has any developing embryos (Laughton, Fan & Gerardo, 2014). Parthenogenetic reproduction in aphids involves live birth, so extensive development occurs within the mother’s abdomen. Aphids have two ovaries that are composed of several ovarioles that carry many embryos at a time to the reproductive opening (Miyazaki, 1987). If an aphid is reproductive, these embryos can be clearly observed in the ovaries through a low-magnification microscope. However, if an individual is post-reproductive, then there will be no embryos in the ovaries.

A third, novel method is also possible. Pea aphids, including embryos, have black to red-pigmented eyes. Eyespots of developing embryos can often be seen within the intact abdomen of live parthenogenetic mothers (Schmidtberg & Vilcinskas, 2016). If these spots are consistently seen in reproductive individuals, but not the post-reproductive, this offers a third method of evaluating reproductive status.

We kept every focal individual in a clip cage to determine when she stopped reproducing, then dissected to determine if she had depleted her stock of embryos. After gathering data on the first 19 individuals, we observed that the eyespots of embryos were often visible through the body wall of reproductive adults. For each of 38 further focal individuals we assessed whether eyespots were visible inside each abdomen. This was done after caging but before dissection. We blinded the results between each method, to ensure independence of results. Each method, and blinding procedures, are described in greater detail below.

### Clip cage method

We made clip-cages by cleanly cutting two rings of foam pipe-insulator (outer diameter of 4 cm, inner diameter of 2.5 cm, and height of 1 cm). One ring had 1 mm meshed fabric glued to it so the aphid could breathe but not escape (Fig. 2). We placed the mesh-covered ring on top of a leaf with the aphid inside and attached it to another ring placed under the leaf. Attachment was by means of three 25 mm long staples pressed into both foam rings from the sides, so as not to damage the leaf. When attaching each clip cage, we were careful not to leave any gap (between the leaf and the cage) through which the aphid could escape.

We isolated focal individuals in clip cages (one aphid per cage, one cage per plant). Every two days, we removed all nymphs from each cage and resealed the adult in the clip cage. If the leaf was damaged or wilting we moved the clip cages to a new leaf. When we found no offspring within the clip cage for two checks (four days) in a row, that individual was recorded as demographically post-reproductive.

### Dissection method

After three weeks in clip cages, roughly half of individuals (totaling 26 of 57) had been labelled as demographically post-reproductive. At that point each individual was isolated in a covered aliquot tube for further study. Blinding (see below) ensured that each individual’s status was recorded but not known during further examination.

**Figure 3:**
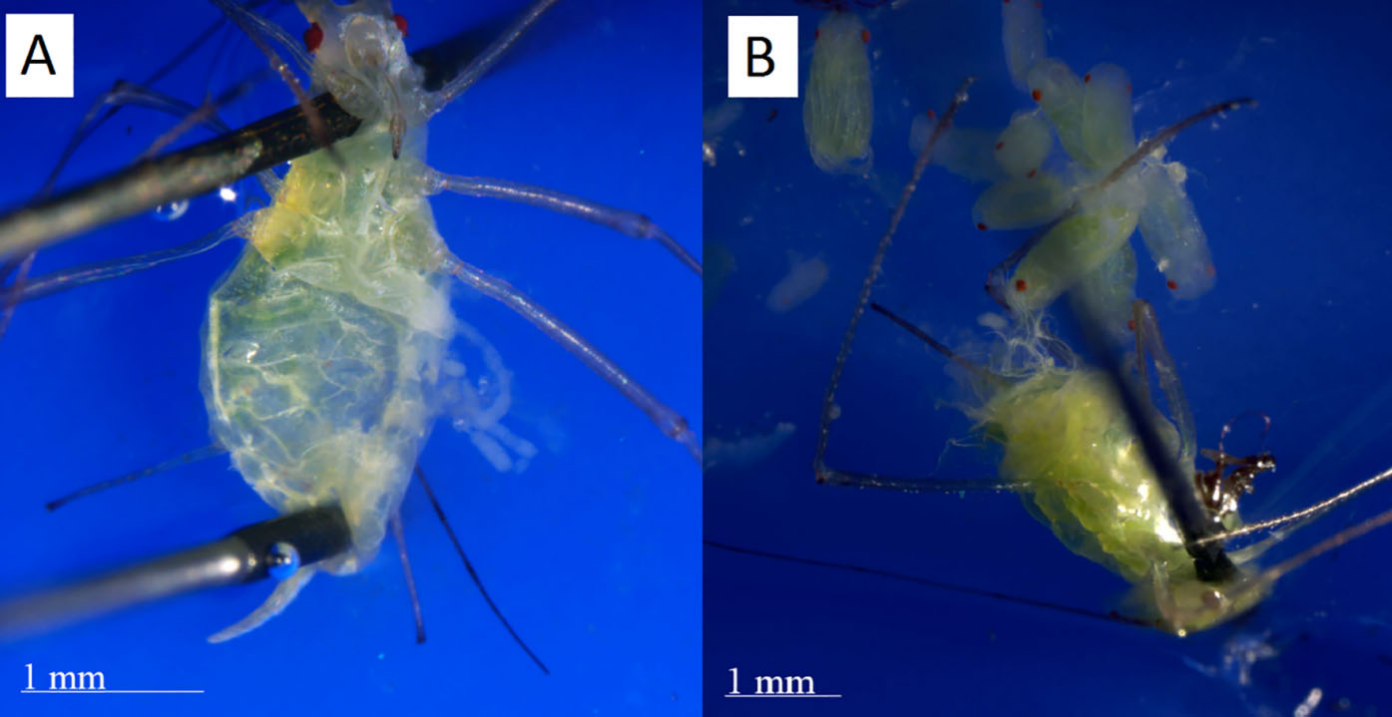
Dissections of post reproductive and reproductive aphids. (A) The post-reproductive aphid’s ovaries, spread to the right, are empty. (B) The reproductive aphid has many embryos in her ovaries, which have been spread out of the abdomen for inspection. Photographs by Erik Saberski and Julia Diamond.

To determine if each individual was physiologically post-reproductive, we dissected her under a microscope (Leica Ez4) at 20x magnification to look for embryos in her ovaries. We placed the aphid supine on a rubber tray and anchored her by carefully piercing her head with a size 000 dissecting pin. If necessary, the posterior edge of the abdomen was also pinned down. Next, we peeled the venter of the abdomen off using the tip of another pin or fine forceps (shown in Fig. 3). We observed the ovaries to see if any embryos remained within the mother (Laughton, Fan & Gerardo, 2014). Where additional visual clarity was needed, we applied a small drop of phosphate-buffered saline solution to the opened abdomen. Embryos could be easily observed if they were present. An individual was marked as physiologically post-reproductive if she had no embryos.

### Eyespot method

**Figure 4:**
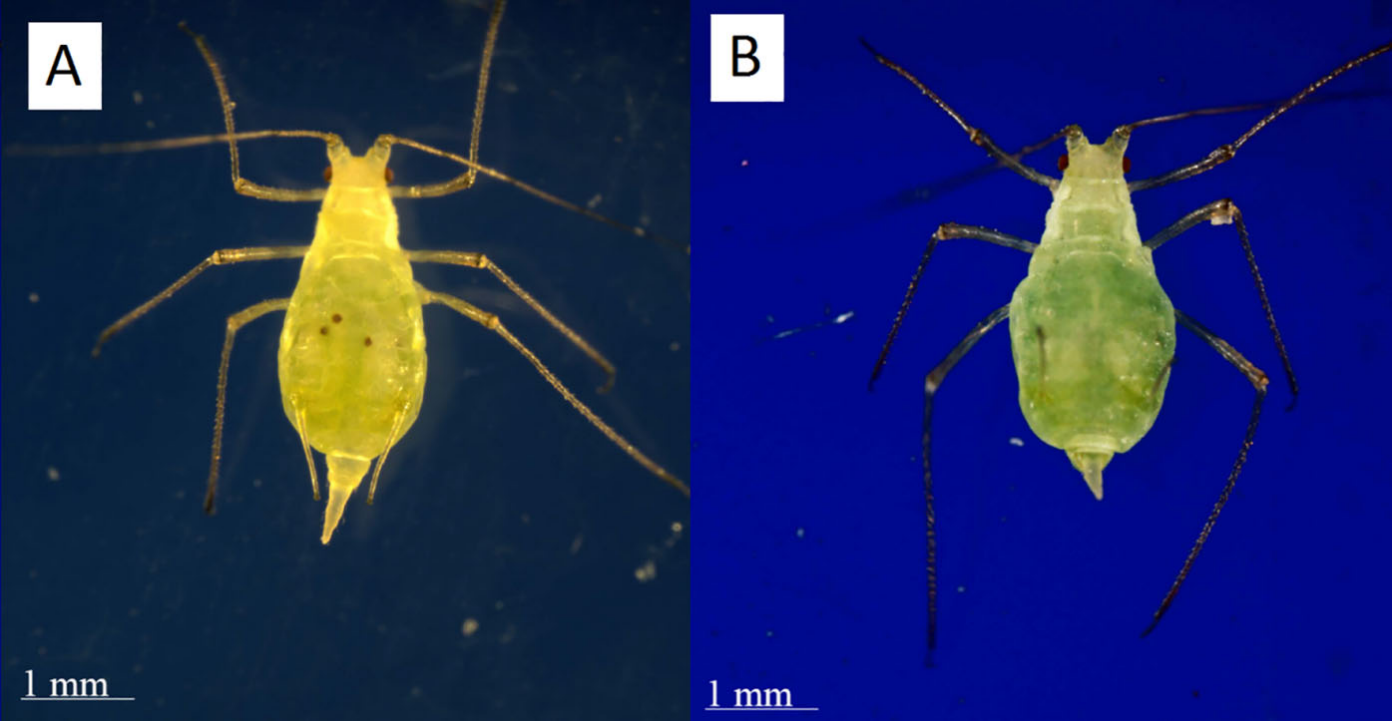
Distinguishing post-reproductive individuals visually. (A) Reproductive adults have eyespots of developing embryos visible within their abdomens. (B) Post-reproductive individuals do not. While post-reproductive individuals’ abdomens and cauda often appear more asymmetrical and flacid than those of younger adults, this difference is not diagnostic. Photographs by Erik Saberski and Julia Diamond.

To check for eyespots we removed each aphid from her test tube and looked at her both supine and prone under 20x magnification (Fig. 4). We found that immobilizing the individual was not necessary since it did not lead to a change in diagnosis.

**Figure 5:**
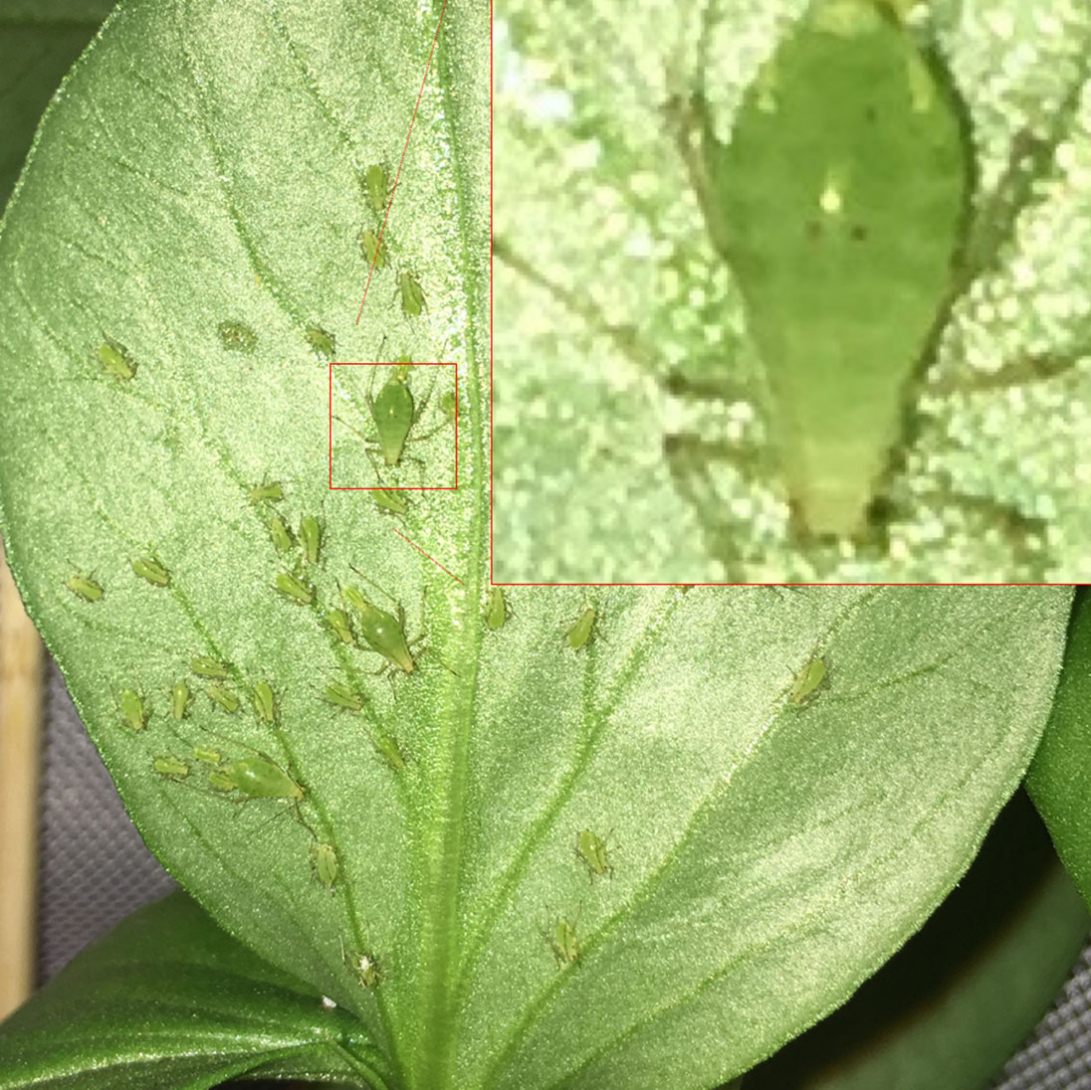
A pea aphid colony containing three reproductive adults. These three adults are all considered to be within the center of the colony, as they have adjacent aphids on a least three of four sides. All three were diagnoses as reproductive, as digital zooming (inset) revealed visible embryonic eyespots within their abdomens. Photograph by Nathaniel Henneman.

### Blinding and data handling

In order to make results from our three methods experimentally independent of each other we used the simple blinding method of placing each individual in an aliquot tube after her removal from the clip cage, writing her data on her tube, and then covering the tube in aluminum foil. After the eyespot method, we wrote our data on the aluminum foil then covered it with another layer of foil. Results from dissections were written on this final layer of foil. At the end of the experiment these layers were peeled off and the data tabulated for comparison. Data analysis consisted of tallying the number of cases in which each method agreed with the others.

### Examining colony structure

In order to examine whether post-reproductive individuals are more prone to move away from the center of a pea aphid colony than are reproductive individuals, we established seven colonies, each on a separate fava bean plant. Previously uninfested plants were covered in fine mesh bags (with zippers for experimenter access) and inoculated with six aphids each: two nymphs, two reproductive adults, and two post-reproductive adults (all adults reared singly in clip cages and identified to stage using the eyespot method). All six were placed on the top of the largest leaf of each plant. These aphids were then given five to seven days to establish themselves on the plant, reproduce, and organize themselves into a colony. At the end of this time, the bag was unzipped and the plant thoroughly examined for aphids. All aphids on the plant were photographed in situ with a 4.5 mm 12 Megapixel iPhone 6S camera and the colonies left intact for further study. Preliminary testing showed that photographs taken in this way consistently revealed intraabdominal eyespots where present. From these photographs, each aphid was classified as being a nymph, reproductive adult or post-reproductive adult. Nymphs were distinguished from adults by observing their cauda (tail-like appendage), and reproductive and post-reproductive adults were distinguished using the eyespot method. Digitally zooming into the photographs was often necessary to observe the eyespots. Separately, each aphid was classified as being internal to the colony, at the edge of the colony, or not on the same leaf as the colony. All plants had a single clearly identified colony and most had a few individuals on other leaves. The ‘center’ was defined as in the colony, surrounded by other aphids on at least three of four sides. The ‘edge’ was defined as any location on the same leaf as the colony, but not surrounded by other aphids on at least three of four sides.

## Results

### Identifying post-reproductive individuals

In every case, all methods agreed on each individual’s reproductive status. Of the 57 adult apterous aphids that were kept in clip cages, we found 31 to be reproductive and 26 to be post-reproductive. All 57 of these were dissected and 38 were examined for internal eyespots. Of these 38, 23 had spots, and 15 had none. All individuals scored as demographically reproductive were also physiologically reproductive, and all of these examined had embryonic eyespots visible. Likewise, all post-reproductive individuals were identified as such across methods.

### Colony structure

As previously reported, reproductive individuals were mostly positioned within the colony (12 of 20 individuals) (Fig. 5) with the remainder appearing at the edge of the colony (5 of 20). In contrast, post-reproductive individuals were mainly at the edge of the colony (11 of 16) or outside the colony (4 of 16) with only one post-reproductive individual observed within the colony. On all seven plants, more post-reproductive individuals were found on the edge or out of the colony, than within the colony. In contrast, on every plant at least half of the reproductive individuals were found in the colony. Full data, by plant, are given in Table 1. Pooling across plants, Fisher’s Exact Test (fisher.test in package *stats*, R 3.3.1, R Development Core Team (2016)) allows us to reject the null hypothesis (two sided p = 0.002) of independence of rows (counts of reproductive and post-reproductive individuals) and columns (In, Edge, or Out locations).

**Table 1:** Pea aphid composition by plant, location and life stage. Each of the pea aphids on seven plants, tabulated by location and status as a nymph, reproductive adult or post-reproductive adult. On all seven plants, reproductive adults are more likely than post-reproductive adults to be located at the center of the colony.

Plant	Life stage	Center	Edge	Outside
1	Nymph	8	1	0
	Ri	1	1	0
	PRi	0	0	1
2	Nymph	22	12	0
	Ri	3	0	0
	PRi	0	1	0
3	Nymph	22	12	2
	Ri	2	1	1
	PRi	0	2	0
4	Nymph	14	10	0
	Ri	1	0	0
	PRi	0	1	1
5	Nymph	30	21	3
	Ri	2	1	1
	PRi	0	3	1
6	Nymph	34	19	0
	Ri	2	1	1
	PRi	1	2	0
7	Nymph	13	11	0
	Ri	1	1	0
	PRi	0	2	1

## Discussion

Our fast, easy, accurate, and non-invasive method for identifying post-reproductive pea aphids reveals that they, in contrast to reproductive adults, are generally positioned at the edges of or away from colonies.

### Identifying post-reproductive individuals

All three methods we tested identified the same parthenogenetic pea aphids as post-reproductive. However, there are significant logistical differences between these methods. The clip cage method requires waiting at least four days after reproduction ceases before an individual is certainly post-reproductive. While we observed no cases in which an individual had no offspring for four days but later reproduced (even though most of our post-reproductive individuals were kept alive for additional days before dissection) we did observe four interbirth intervals of two days. Also, this method is comparatively labor intensive because caging each individual aphid took about three minutes each time and cleaning off any offspring took an additional minute per individual at each check. During checks, care must be taken to avoid allowing the focal aphids to escape. This method is effective at creating a population of post-reproductive individuals for study, but as a diagnostic method requires some days of waiting during which some individuals may die. In contrast, dissecting each individual (Fig. 3), while fatal, gives clear results after five minutes of work and no wait time. Training to effectively and quickly perform dissections takes about an hour, and interpretation is clear; an adult with no embryos is post-reproductive. Laughton, Fan & Gerardo (2014) report that some post-reproductive individuals retain up to eight putrefying embryos. While we did not find this in our population, those using this method should be aware that dissections of post-reproductive individuals may yield a few embryos, but these will be clearly dead. These two methods of determining post-reproductive status of parthenogenetic individuals would likely be effective on any species of aphid since they all have similar reproductive morphologies (Miyazaki, 1987), although times and logistics will vary among species.

The eyespot method is the fastest, easiest, and least invasive method of determining the reproductive state of an aphid in our population. Since sexual reproduction involves the laying of eggs containing early stage embryos unlikely to yet have eyespots, this method likely wouldn’t work beyond the parthenogenetic context. Examination of 10 winged parthenogenetic individuals in our lab revealed eyespots visible in the abdomens of all 10, implying that wingedness does not hinder the use of our method. Red pigment in the mother’s body wall could potentially make seeing the colored eyespots of her embryos more difficult. While we have no data on red morph individuals, review of some published images of red pea aphids reveal that in at least some of them (Savage, 2010) spots consistent with embryonic eyes are visible within maternal abdomens. In short, for species of aphids where embryonic eyespots are visible inside the mother, this method is likely to be faster (well under one minute per individual) and less invasive than either pre-existing method for identifying post-reproductive individuals. We further note that individuals that have molted into adults only hours earlier show clear embryonic eyespots, such that pre-reproductive adults are unlikely to be mistaken for post-reproductive. Post hoc, this method seems efficient in quantifying approximately how many developing embryos were in an individual’s ovaries, but this will require further study.

### Colony structure

Post-reproductive pea aphids, unlike younger adults, were most often found at the edges of the colony or on a different leaf entirely. Several mechanisms may explain this difference. Possible adaptive explanations include these post-reproductive individuals helping their kin in the colony by acting as guards or sentinels of some kind, or simply making space within the colony for reproductive adults and nymphs, both of which have higher reproductive value to the clone. Pea aphids, like many other aphids, use an alarm pheromone, and post-reproductive sentinels could potentially alert the colony to an approaching predator or parasite. Non-adaptive explanations, such as decreasing mobility and sensory acuity in senescent adults causing them to separate from the colony, are also possible.

Post-reproductive individuals are found in the most exposed locations in both pea aphids and Japanese gall aphids (*Quadrartus yoshinomiyai*). It may be that this pattern is found more broadly across aphids. If so, post-reproductive aphids of many species may play some important role in the life of the colony.

### Developing pea aphids as a model system for studying adaptive post-reproductive life

One of biology’s most successful strategies for understanding the evolution of phenotypic traits is to have a tractable experimental population in which that trait occurs and can be manipulated. Such a system for studying the evolution of adaptive post-reproductive life would be (a) an organism that frequently experiences post-reproductive life in the wild, (b) where post-reproductive life has a strong positive selective value, (c) in which post-reproductive individuals are non-invasively and easily identifiable, (d) that is easy to keep and study in the laboratory, (e) that is available to experimenters all over the world, and (f) which has already been studied by a wide range of other biological disciplines. While there are several candidate species proposed to have (a) and (b), and most common laboratory animals have (d), (e) and (f), we as yet have no organism that combines all of these desirable properties. *Q. yoshinomiyai,* a Japanese gall aphid, is a strong contender for eventually having all of the properties. Our results suggest, but do not prove, that pea aphids are also a likely candidate.

Whether field population of pea aphids experience significant post-reproductive life is a key question. The measure necessary to answer that question is Post-reproductive Representation (PrR) which is equal to the proportion of the mean individual’s adult life which is lived after the age at which members of her (or his) population have completed most of their fertility. PrR and significance testing thereof are introduced and derived by Levitis and Bingaman Lackey, and its evolutionary use expanded upon by Levitis, Burger & Lackey (2013). Populations of women generally have PrR values between 0.4 and 0.8, greater than any other species studied to date. Wild primates display PrRs of < 0.1. PrR in one laboratory population of pea aphids reared at 14.8 °C was 0.37 (Campbell & Mackauer, 1977). Ongoing research in our lab aims to determine whether, and under what circumstances, significant PrRs can be observed in wild pea aphid populations.

## Conclusion

The study of the evolution of post-reproductive life has long focused on humans, alone or in a comparative context. Shifting focus from understanding humans to understanding the evolution of post reproductive life allows for, perhaps requires, examination of post-reproductive life in species that are in many ways dissimilar from humans. Aphids, because of their reproductive physiology, complex life-cycles and sociobiology are appealing targets for investigation of potentially kin-selected post-reproductive life stages. We have shown that post-reproductive pea aphids can easily, accurately, and non-invasively be identified using the eye-spot method, and that these individuals place themselves distal to the rest of the colony. This raises several key questions about post-reproductive life in pea aphids. Is it adaptive, or merely the result of demographic stochasticity? How often do post-reproductive individuals occur in populations outside of the laboratory, and do they behave in the field as we have observed in the lab? Do they help to protect younger kin from predators or parasites? Do they aid in the growth or reproduction of kin? Do they impose costs on kin? In what respects (other than placement and reproduction) does the behavior of post-reproductive individuals differ from that of reproductive? Do many species of aphids have adaptive post-reproductive survival? Answering these questions may advance the study of kin-selected post-reproductive life stages from being largely observational and correlational to having an established experimental model.

## Supplemental Information

10.7717/peerj.2631/supp-1 Supplemental Information 1 Raw data collected for each method tested. The experiment was done in five sessions, each represented by a different box. Each box notes the day of the experiment for that given session when analyzing the clip-cage method. The presence of nymphs was marked every two days for the clip-cage method. If nymphs were present, the corresponding row was marked with “Nymphs” and if no nymphs were present, it was marked with “0.” Below each box, the results for both the clip-cage method and dissection method are labeled with either “R” for reproductive, or “P” for post-reproductive.

## Additional Information and Declarations

### Data Deposition

The following information was supplied regarding data availability: The raw data has been supplied as Supplemental Dataset Files.
